# Ultrasonically Assisted Single Point Diamond Turning of Optical Mold of Tungsten Carbide

**DOI:** 10.3390/mi9020077

**Published:** 2018-02-12

**Authors:** Zhanjie Li, Gang Jin, Fengzhou Fang, Hu Gong, Haili Jia

**Affiliations:** 1 Tianjin Key Laboratory of High Speed Cutting and Precision Machining, Tianjin University of Technology and Education, Tianjin 300222, China; mtydhghd@126.com (Z.L.); hljia@tute.edu.cn (H.J.); 2 State Key Laboratory of Precision Measuring Technology & Instruments, College of Precision Instrument and Opto-Electronics Engineering, Tianjin University, Tianjin 300072, China; fzfang@tju.edu.cn (F.F.); gonghu@tju.edu.cn (H.G.)

**Keywords:** tungsten carbide, lens mold, single point diamond turning (SPDT), ultrasonic vibration, Ductile regime, surface quality, wear

## Abstract

To realize high efficiency, low/no damage and high precision machining of tungsten carbide used for lens mold, a high frequency ultrasonic vibration cutting system was developed at first. Then, tungsten carbide was precisely machined with a polycrystalline diamond (PCD) tool assisted by the self-developed high frequency ultrasonic vibration cutting system. Tool wear mechanism was investigated in ductile regime machining of tungsten carbide. The cutter back-off phenomenon in the process was analyzed. The subsequent experimental results of ultra-precision machining with a single crystal diamond tool showed that: under the condition of high frequency ultrasonic vibration cutting, nano-scale surface roughness can be obtained by the diamond tool with smaller tip radius and no defects like those of ground surface were found on the machined surface. Tool wear mechanisms of the single crystal diamond tool are mainly abrasive wear and micro-chipping. To solve the problem, a method of inclined ultrasonic vibration cutting with negative rake angle was put forward according to force analysis, which can further reduce tool wear and roughness of the machined surface. The investigation was important to high efficiency and quality ultra-precision machining of tungsten carbide.

## 1. Introduction

Today, there is an increasing demand for aspheric lenses with the rapid development of the optical industry. The precision molding technique has become an important method for making optical components because of its high efficiency and high precision [1,2]. To undergo the process environment of high temperatures (400–800 °C) and large forces (1–10 kN) in the precision molding technique, the mold material should be heat-resistant and hard [3]. So far, ceramics have become the most promising material for precision molding, especially superfine tungsten carbide [4,5,6]. The molds for precision molding are typically ultra precision ground technique with a subsequent polishing [7,8,9], which is a process time-consuming process with low reproducibility. On the other hand, ultra-precision diamond turning is a mechanical manufacturing process that can achieve sub-nanometer level surface finishes (below 5 nm Ra) and sub-micrometer form accuracies (below 300 nm) on complex geometries [10]. Therefore, making optical molds with high efficiency, low/no damage and high precision can be realized by ultra-precision diamond turning [11]. The ultrasonic assisted ultra-precision diamond turning has already proved its potential for machining hard-to-cut materials such as steel [12], Co-Cr-Mo alloy [13], single-crystal silicon [14] and glass [15,16]. These publications demonstrate that ductile mode processing of tungsten carbide is possible [17,18,19]. 

In this paper, tungsten carbide will be ultra-precision machined using (Single Point Diamond Turning) SPDT assisted by a self-developed high frequency ultrasonic vibration cutting system. Critical depth of cut and wear mechanism will be investigated in ductile regime machining of tungsten carbide. Some phenomena and problems in the process will be analyzed, and then corresponding solutions will be put forward. This work has the extensive applicability and practical significance for ultra-precision machining tungsten carbide and optical molding industry.

## 2. Experimental Preparation

### 2.1. Ultrasonic Vibration Cutting System

**Figure 1:**
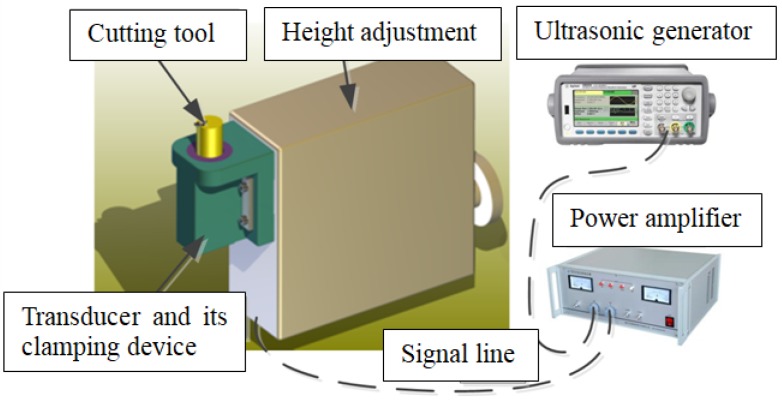
Schematic diagram of the ultrasonic vibration cutting system used in this experiment.

As shown in Figure 1, the ultrasonic vibration cutting system used in this experiment consists of ultrasonic generator, power amplifier, horn, transducer and its clamping device, cutting tool and integrated micro height adjustment. A digital-tracking-mode ultrasonic generator with working frequency scope between 20 and 100 kHz was selected in order to ensure stable frequency and vibrating amplitude in the cutting process.

### 2.2. Modal Analysis of the Horn Using Finite Element Method

In order to avoid interference with tool setting gauge, ultrasonic horn made of quenched alloy steel was designed to an upward cutting end and a fixed end with vibration isolation groove. In order to analyze natural frequencies of the horn before experiment, a finite element analysis was carried out. 

**Figure 2:**
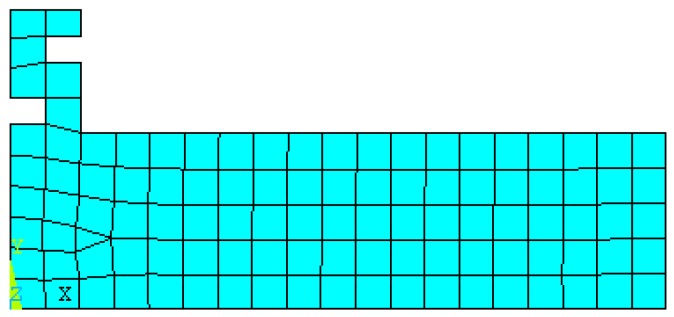
Axial section and grid partition.

**Figure 3:**
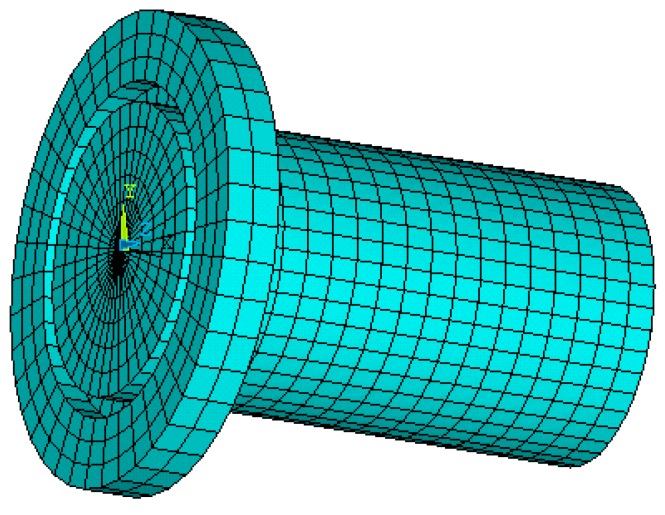
Horn model and grid partition.

In the finite element analysis (FEA), material attributes were set as follows: young modulus was set to be 206 GPa, poison ratio was set to be 0.27, and mass density was set to be 7900 kg/m^3^. Plane 42 was chosen as the surface element and Solid 95 was chosen as the body element for the finite element model. The boundary condition was assumed to be isothermal [20]. Mesh tool was selected to control mesh size. Grid partition of the axial section and horn model were shown in Figure 2 and Figure 3, respectively.

**Figure 4:**
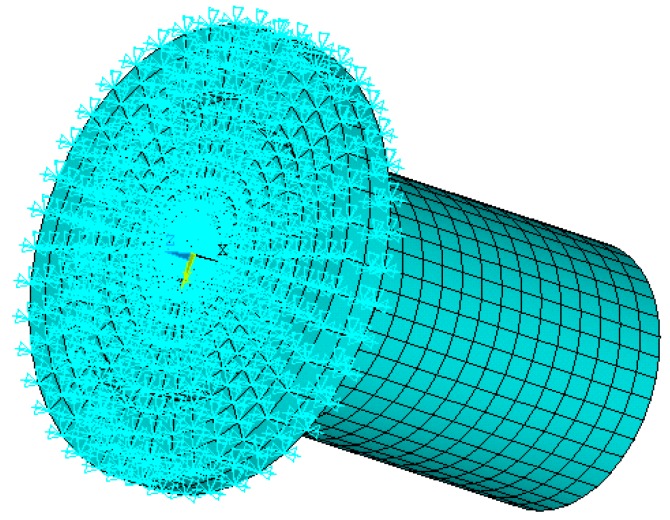
Imposing constraints on upper-end of the horn.

Modal analysis was set for the FEA and subspace was set for the extraction method. The extraction scope of frequencies was 30–90 kHz and the modal order number was set to be 10. Displacement constraints were imposed on upper-end of the horn spatial model, as shown in Figure 4.

**Figure 5:**
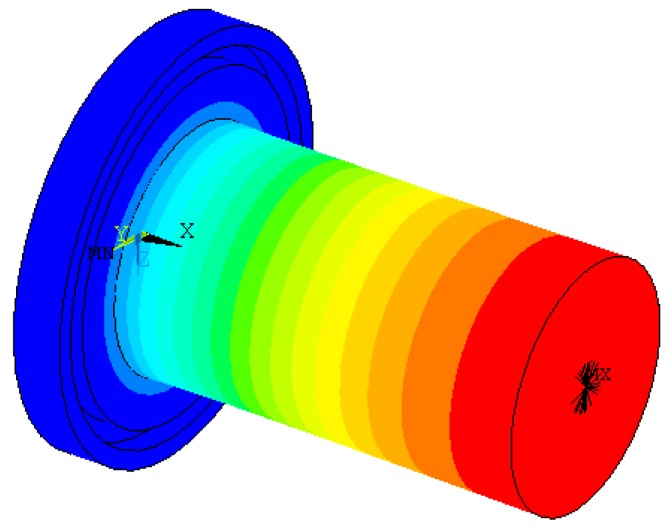
Vibration mode shape of cylindrical horn at frequency of 67.533 kHz.

**Figure 6:**
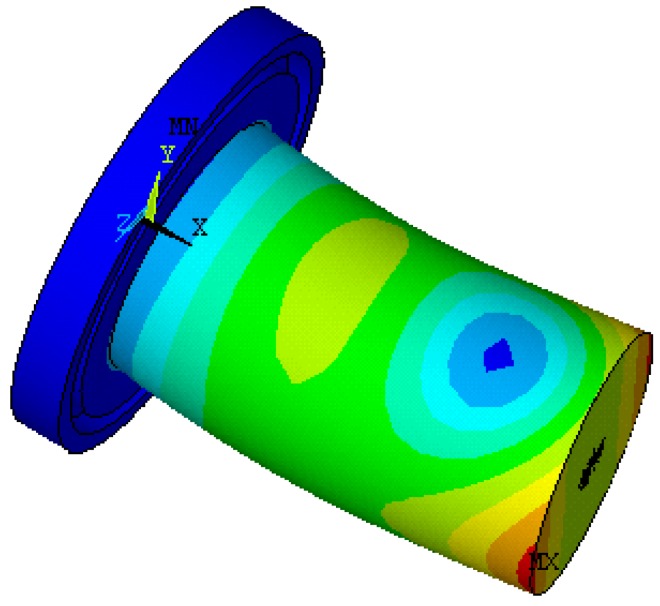
Vibration mode shape of cylindrical horn at frequency of 38.198 kHz.

Corresponding natural frequencies of the horn between 30 kHz and 90 kHz when resonance occurred are shown in Table 1. By observing vibration mode shapes of the horn, we know that axial resonance occurred at a frequency of 67.533 kHz. Vibration mode shape of the horn at frequency of 67.533 kHz is shown in Figure 5. No axial resonance occurred at other frequencies. The vibration mode shape of the horn at a frequency of 38.198 kHz is shown in Figure 6. 

A sandwich transducer was fabricated using PZT8 piezoelectric ceramics, and the electrode slices was fabricated using beryllium copper.

### 2.3. Performance Testing of the Ultrasonic Vibration Cutting System

The no-load test results of the developed ultrasonic vibration cutting system showed that the cutting end reached resonance at a frequency of 64.769 kHz. The experimental result is in good agreement with the FEA result. The laser interference vibration measuring system (SIOS, SP-S, SP-S 120/500, SIOS MeBtechnik GmbH, Ilmenau, Germany) was adopted to track the amplitude of the cutting end. The corresponding amplitude between 1 and 14µm was measured at the voltage range of 0–400 V. 

### 2.4. Experimental Condition and Plan

J05 tungsten carbide with a diameter of 16 mm was used in this experiment. Material properties of J05 were shown in Table 2. A precision cutting process with PCD tool (tip radius 1 mm, Shenzhen Yuhe Diamond Tools Co., Ltd., Shenzhen, China) was arranged at first due to lower surface smoothness of the specimen. Then, a natural single crystal diamond tool was used to ultra-precision cutting from the operator to the center of the workpiece along the *X*-axis direction. Both processes were performed with lubrication of light mineral oil (isopar H).

**Figure 7:**
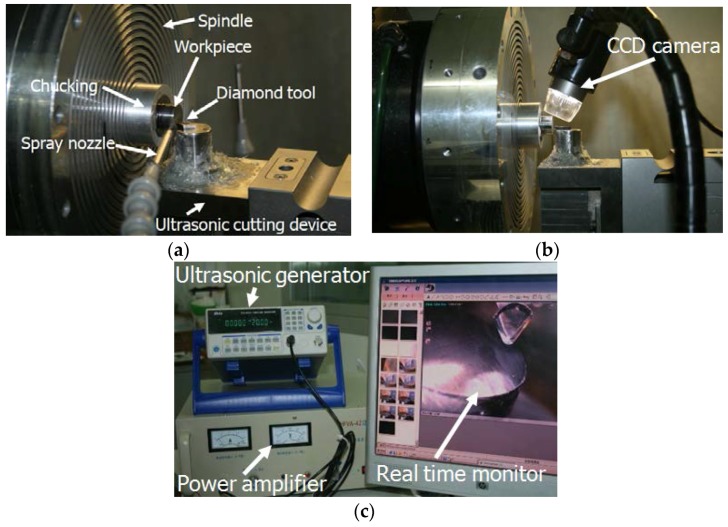
Schematic of the experiment system; (**a**) Cutting end; (**b**) Tool setting assisted by real time monitoring; (**c**) Ultrasonic generator and power amplifier.

As shown in Figure 7, the self-developed high frequency ultrasonic vibration cutting system was amounted on an ultra-precision lathe (Nanotech 250UPL, Moore Nanotechnology System, LLC, Keene, NH, USA). The tool setting and cutting processes were assisted by a real time monitoring system.

### 2.5. Test Method

A white light interferometer was used to measure the surface topography and roughness of the machined workpiece. A 3D digital microscope was used to observe the topography of diamond tool wear.

## 3. Results and Analysis

### 3.1. Precision Machining Using PCD Tool and Results

The selected process parameters were as follows: the spindle rotation speed was set to be 100 rpm, the feed rate was set to be 1 mm/min, the depth of cut was set to be 3 µm, the frequency for ultrasonic vibration cutting system was set to be 65 kHz and the vibrating amplitude adjusted by power amplifier was set to be 2 µm.

**Figure 8:**
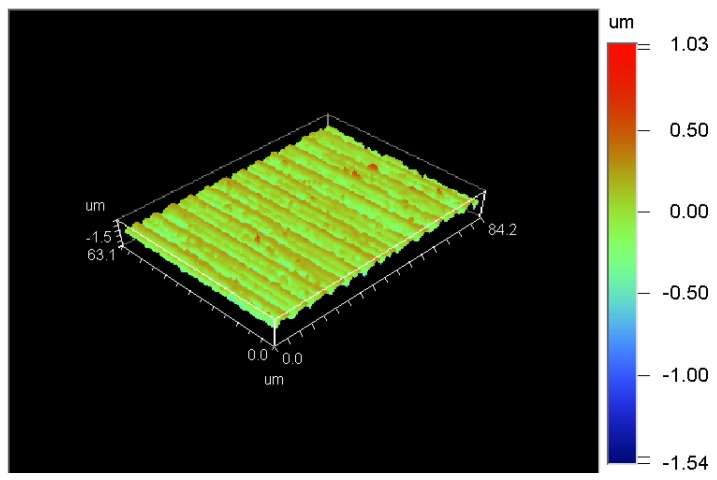
Surface topography and roughness of the workpiece machined by (polycrystalline diamond) PCD tool (Ra 173.56 nm).

The surface roughness of the machined workpiece is 173.56 nm and the surface topography, which reached the requirement for ultra-precision machining, is shown in Figure 8.

### 3.2. Ultra-Precision Machining Using Natural Single Crystal Diamond Tool

To obtain nano-scale surface roughness, a diamond tool with a larger tip radius of 1.5 mm was adopted according to the geometrical factor. The selected process parameters were as follows: the spindle rotation speed was set to be 50 rpm, the feed rate was set to be 0.01 mm/min, the depth of cut was set to be 3 µm, the frequency for ultrasonic vibration cutting system was set to be 65 kHz and the vibrating amplitude adjusted by power amplifier was set to be 2 µm.

**Figure 9:**
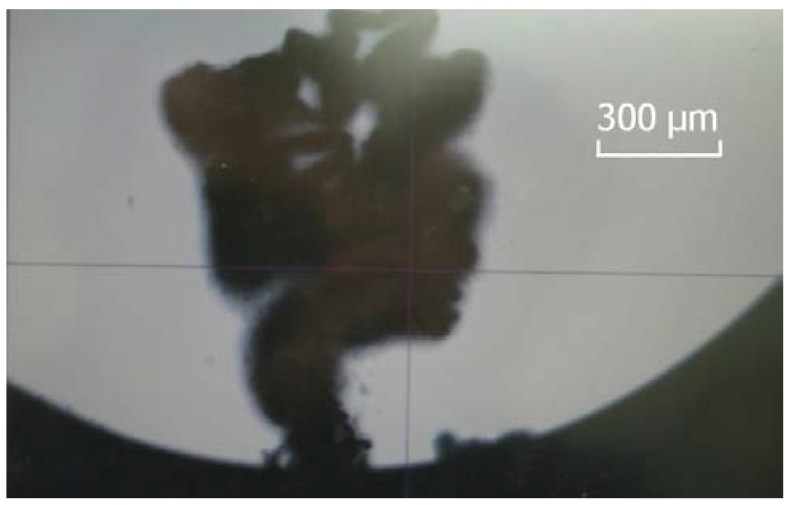
Continuous and ribbon-like chip.

To ensure cutting in the ductile region, the trial cutting method was adopted to decide the cutting depth. As shown in Figure 9, a continuous and ribbon-like chip appeared at the cutting depth of 200 nm. Thus, the optimal cutting depth was determined.

**Figure 10:**
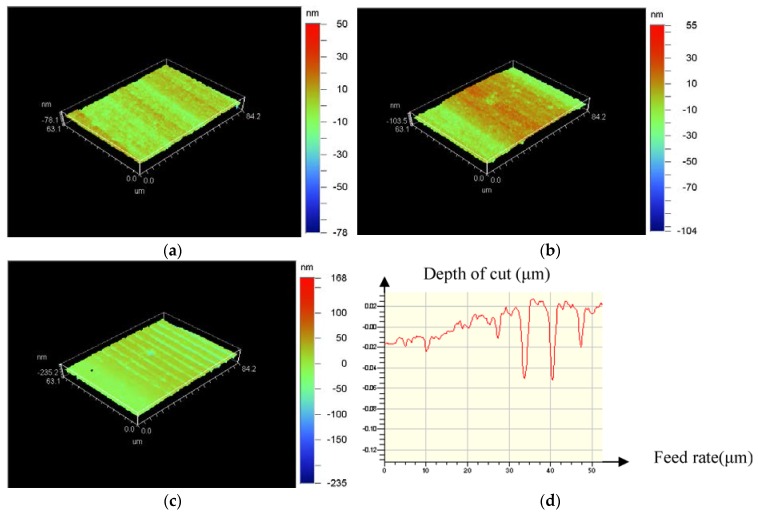
Back-off phenomenon of diamond tool and surface topography of the machined workpiece; (**a**) Surface roughness (Ra 4.72 nm) near the edge of workpiece; (**b**) Surface roughness (Ra 9.85 nm) before back-off phenomenon; (**c**) Surface roughness (Ra 17.3 nm) at back-off phenomenon; (**d**) Two-dimensional graph of back-off phenomenon.

A back-off phenomenon happened when displacement distance of the diamond tool is 2 mm along *X*-axis. The corresponding surface topography of the machined workpiece is shown in Figure 10.

Figure 10 showed that surface roughness of 4.72 nm was obtained near the edge of the workpiece when the diamond tool cut into the workpiece. Then, the roughness was changed to 9.85 nm after a longer cutting distance. Subsequently, the cutting process cannot proceed after back-off phenomenon occurred, as shown in Figure 10c,d.

**Figure 11:**
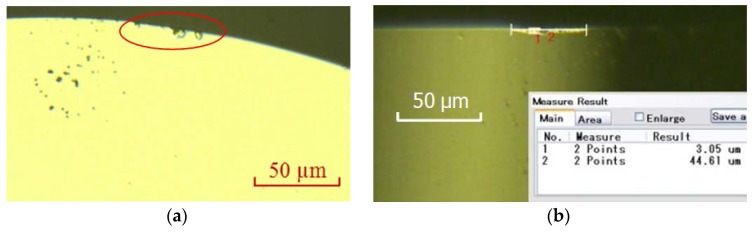
Wear of diamond tool with tip radius of 0.5 mm after cutting along *X*-axis with a distance of 1 mm (wear land width: 3.05 µm, wear land length: 44.61 µm); (**a**) Wear of rake face; (**b**) Wear of rear face.

**Figure 12:**
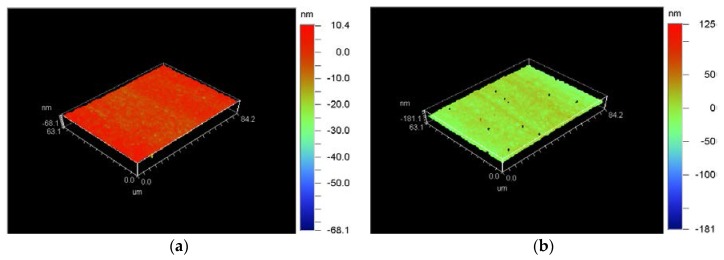
Surface topography of the machined workpiece after cutting along *X*-axis with a distance of 1 mm by a diamond tool with tip radius of 0.5 mm; (**a**) Ra 2.55 nm when diamond tool cutting in a very short distance; (**b**) Ra 8.85 nm when diamond tool cutting in a distance of 1 mm.

The reason for the back-off phenomenon was assumed to be the hardness of tungsten carbide and the tool tip radius. The stress put on the diamond tool was so strong that the screw was loosened due to the longer contact between tool tip and workpiece. To reduce the stress, a diamond tool with a tip radius of 0.5 mm and lower spindle rotation speed were adopted. Figure 11a showed an obvious chipping on the tool edge (in the red elliptic mark). In Figure 11b, some small grooves were found in the wear land. So, wear mechanisms of the single crystal diamond tool were mainly abrasive wear and micro-chipping according to Figure 11 as well as surface topography of the machined workpiece, as shown in Figure 12. The surface roughness of the machined workpiece was Ra 2.55 nm when diamond tool cutting in a very short distance, but gradually rose to Ra 8.85 nm due to the longer cutting distance and tool wear. Surface quality was so good that no micro crack was found in the surface topography of the machined workpiece.

### 3.3. Discussion

**Figure 13:**
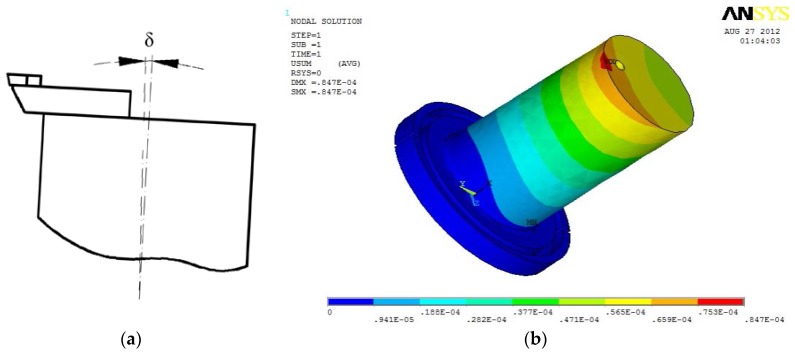
Force analysis of ultrasonic horn; (**a**) Schematic diagram of force analysis; (**b**) Finite element analysis of the force on horn.

**Figure 14:**
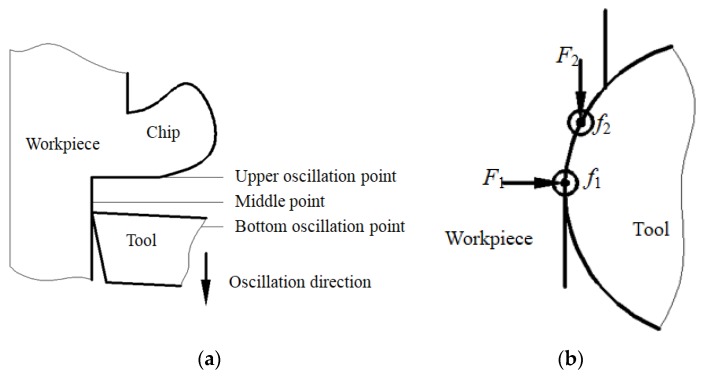
Schematic diagram of force analysis at the stage of diamond tool withdrawing; (**a**) Front view; (**b**) Top view.

From the above experimental results, the strong stress and friction between tool tip and workpiece easily gave rise to an inclined angle *δ* of ultrasonic horn, which changed 0° of rake angle to a positive rake angle, as shown in Figure 13. In the cutting direction, cutting force *F*_1_ and friction force *f*_1_ existed between flank surface of diamond tool and machined surface of workpiece at the stage of withdraw of the diamond tool from the chip. At the same time, in the feed direction, cuttingforce *F*_2_ and friction force *f*_2_ existed between the flank surface of the diamond tool and the transitional surface of the workpiece when the diamond tool left the chip (Figure 14). Therefore, diamond tool micro-chipping happened very easily due to the impact and alternating stress between the diamond tool and the workpiece [21]. 

**Figure 15:**
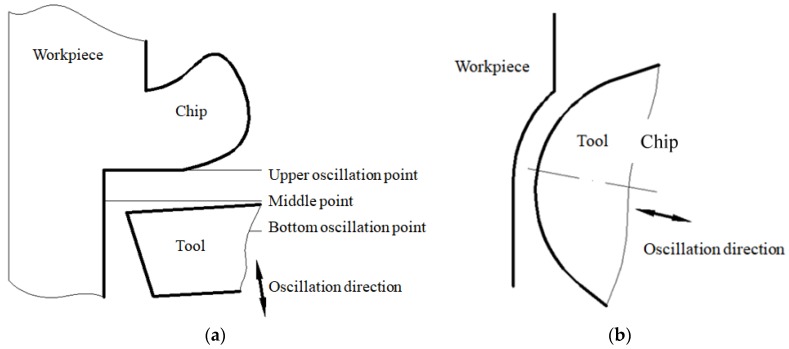
Schematic diagram of inclined ultrasonic vibration cutting with negative rake angle; (**a**) Front view; (**b**) Top view.

In the inclined ultrasonic vibration cutting with negative rake angle, the diamond tool vibrated along the direction, as shown in Figure 15. On one hand, not only inclined cutting can decrease the impact force between diamond tool and workpiece, but also cutting with negative rake angle can enhance the strength of diamond tool. On the other hand, inclined cutting can avoid the contact and friction between the rear face of the diamond tool. At the same time, the separation between the diamond tool and the workpiece can help the cutting fluid reach the tool tip region, which can cool and lubricate the diamond tool completely. In this method, the size of both negative rake angle and included angle between vibrating direction and cutting direction had a great influence on surface roughness and tool wear.

To validate the proposed method and raise machining efficiency, an experiment was conducted under the following conditions: tip radius was 0.5 mm, light mineral oil (isopar H) was selected as lubrication, the spindle rotation speed was set to be 400 rpm, the feed rate was set to be 1 mm/min, the depth of cut was set to be 3 µm, the frequency for ultrasonic vibration cutting system was set to be 65 kHz and the vibrating amplitude adjusted by power amplifier was set to be 2 µm, inclination between rake face and *XOZ* plane was set to be about −5°, inclination between the center line of diamond tool and *Z*-axis was set to be about 5°.

**Figure 16:**
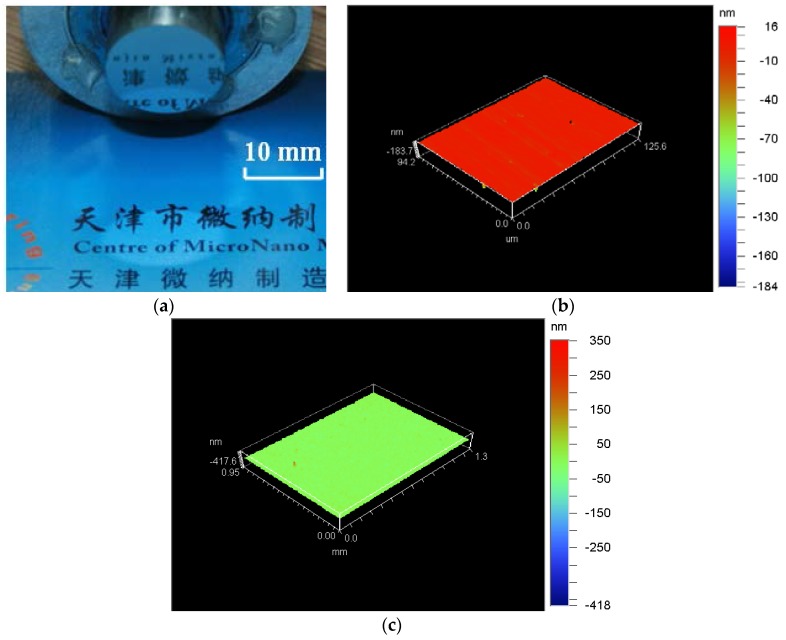
Surface topography of the machined workpiece using the proposed method; (**a**)Photo of the machined workpiece; (**b**) Ra 1.82 nm when diamond tool cutting in a very short distance; (**c**) Ra 6.2 nm when diamond tool cutting near the center of workpiece.

Figure 16 showed surface topography of the machined workpiece using the proposed method. Surface roughness of the machined workpiece has been improved (Ra 1.82–6.2 nm) compared to that of conventional ultrasonic vibration cutting (Ra 2.55–8.85 nm, within a distance of 1 mm).

**Figure 17:**
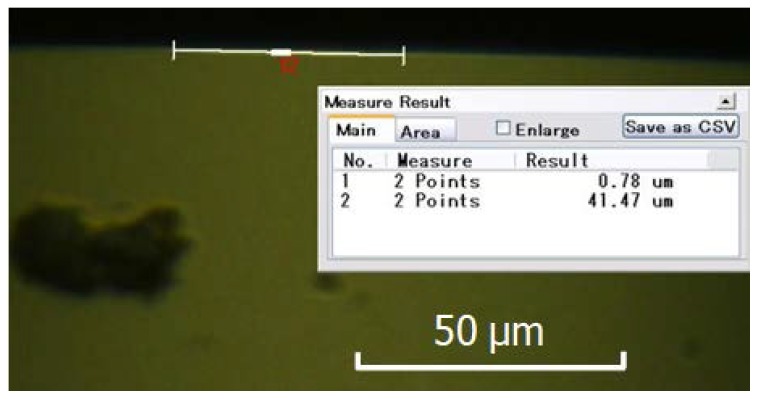
Wear of diamond tool with tip radius of 0.5 mm after cutting along *X*-axis with negative rake angle (wear land width: 0.78 µm, wear land length: 41.47 µm).

Figure 17 showed wear of the diamond tool using the proposed method. Wear has been improved (wear land width 0.78 µm, wear land length 41.47 µm) compared to that of conventional ultrasonic vibration cutting (wear land width 3.05 µm, wear land length 44.61 µm). Tool wear land of single crystal diamond tool was smaller and no obvious chipping was found on the tool edge.

## 4. Conclusions

(1) A high frequency ultrasonic vibration cutting system was developed, and a tungsten carbide was precisely machined with a PCD tool assisted by the cutting system. Tool wear mechanism was investigated in ductile regime machining of tungsten carbide. The cutter back-off phenomenon in the process was analyzed.

(2) Tool wear mechanisms of both PCD and single crystal diamond tool were mainly abrasive wear and micro-chipping under the condition of high frequency ultrasonic vibration. The surface roughness of the machined workpiece gradually increased with the increased diamond tool wear. The size of tip radius had a great influence on diamond machinability of tungsten carbide. A nano-scale surface roughness can be obtained by using diamond tool with smaller tip radius and no defects were found on the machined surface.

(3) A method of inclined ultrasonic vibration cutting with negative rake angle was put forward according to force analysis, which can further reduce tool wear and roughness of machined surface. The above investigation is important to high efficiency and quality ultra-precision machining of tungsten carbide.

## Figures and Tables

**Table 1:** Natural frequencies of the ultrasonic horn.

Order	Natural Frequencies (kHz)
1	35.454
2	38.198
3	38.198
4	67.553
5	79.864

**Table 2:** Material properties of tungsten carbide.

Name	Chemical Composition (Weight Percentage %)	Hardness	Grain Size (μm)	Density (g/cm^3^)
J05	WC: 90 (±0.5%) Co.: 10 (±0.5%)	HRA:91.2–92.0 HRC:76.4–77.2	0.6	14.65
